# Assessing Motivation for Engaging in an Alzheimer's Disease Research Registry

**DOI:** 10.1002/alz70858_099720

**Published:** 2025-12-24

**Authors:** Le'Elle Davis, Reginald Wilburn, Theresa F. Gierzynski, Jennifer Dinh, Kate Hanson, Holly Bunker, Bernardo Flores, Joshua T Fox‐Fuller, Renee Gadwa, Arijit K Bhaumik, J. Scott Roberts, Henry L Paulson, Annalise Rahman‐Filipiak

**Affiliations:** ^1^ Michigan Alzheimer's Disease Research Center ‐ University of Michigan, Ann Arbor, MI, USA; ^2^ Michigan Alzheimer's Disease Research Center, Ann Arbor, MI, USA

## Abstract

**Background:**

Research registries are frequently used to facilitate streamlined and targeted recruitment into studies of Alzheimer's disease and related disorders (ADRD), as they provide investigators with direct access to potential participants who are interested in volunteering for studies. However, there has been insufficient research on what factors might motivate individuals to sign up for participant registries. In the current study, we surveyed participants in the Michigan Alzheimer's Disease Research Center's (MADRC) long‐standing Michigan Neurological Data Set (MiNDSet) research registry to better understand why respondents chose to join the registry, and whether motivations differ by demographic characteristics of the participant.

**Methods:**

Participants (*N* = 540) (age = 71.7±7.7) were sent an electronic survey powered through Research Electronic Data Capture (REDCap) between November 2024 and December 2024. 67% of respondents self‐identified as female, 77% self‐identified as White, 14% self‐identified as Black/African American and 0.5% self‐identified as Other. The survey consisted of 25 questions that aimed to better understand participant identities and experiences, study preferences, and barriers and facilitators to engaging in research.

**Results:**

Most participants reported that advancing ADRD research (86%), benefitting future generations of one's own family (62%), and benefitting society (60%) were the strongest motivators for joining the MiNDSet research registry. Compared with non‐White participants, White respondents were more likely to sign up for the registry because of a recent memory diagnosis (*p* = .023). Non‐White participants were more likely to sign up for the registry to gain access to advice and expertise (*p* = .047) and to learn more about ADRD (*p* < .001). There were no significant differences in motivations for signing up for the registry by sex, age, and education.

**Conclusions:**

Our findings suggest that both altruism (e.g., advancing research) and personal benefit (e.g., access to advice and expertise) were strong motivators for engaging in research. Preliminary results suggest potential differences in motivations for research that may be influenced in part by social factors; these differences may inform more tailored and meaningful recruitment strategies for ADRD research. Future research may also consider whether motivations to participate in research vary by cognitive or socioeconomic status.

